# Molecular Epidemiology and Genetic Evolution of Porcine Reproductive and Respiratory Syndrome Virus in Northern China During 2021–2023

**DOI:** 10.3390/v17010085

**Published:** 2025-01-11

**Authors:** Na Yuan, Zuofeng Yang, Fengxia Lv, Lina Dou, Xiangqing Li, Baokai Zhao, Shishan Dong

**Affiliations:** 1 College of Veterinary Medicine, Hebei Agricultural University, Baoding 071000, China; yuanna0099@126.com (N.Y.); 18768885561@163.com (F.L.); 2 Beijing Daweijia Biotechnology Co., Ltd., Beijing 100085, China; 18832740583@163.com (L.D.); lixiangqing57@163.com (X.L.); 17310632570@163.com (B.Z.); 3 Liaoning Provincial Animal Disease Prevention and Control Center, Shenyang 110015, China; lnsdwfyz@163.com; 4 Henan Muxiang Animal Pharmaceutical Co., Ltd., Zhengzhou 450000, China

**Keywords:** porcine reproductive and respiratory syndrome virus (PRRSV), northern China, molecular epidemiological investigation, genetic evolution, recombination analysis

## Abstract

Porcine reproductive and respiratory syndrome virus (PRRSV), an important pathogen affecting the pig industry, is an RNA virus with high genetic diversity. In this study, 12,299 clinical samples were collected from northern China during 2021–2023 to investigate the molecular epidemiological characteristics and genetic evolution of PRRSV. All samples were screened using qRT-PCR and further analyzed through *ORF5* gene and whole-genome sequencing. The results showed that the positive rate of PRRSV in northern China was 18.42%, and positivity rates were relatively high in spring. The phylogenetic analysis of the *ORF5* gene indicated that the 174 gene sequences were classified as PRRSV-2, predominantly found in Lineage 1.8 (L1.8), Lineage 1.5 (L1.5), and Lineage 8 (L8). L1.8 and L1.5 showed considerable polymorphism at decoy and neutralizing epitopes. Mutations of specific amino acids were present in L1.8 and L1.5 at T- and B-cell epitopes. Moreover, the 27 whole-genome sequences were analyzed. As indicated, 24 of them were exposed to gene recombination, and L1.8 provided the backbone for recombination events. The predominant recombination modes were L1.8 + L8.7 + L1.5/L3, with L1.5 and L3.5 generally yielding GP2~GP6 structural proteins. Recombination hotspots were primarily located within the ranges of 780~2200 (Nsp1~Nsp2), 5400~6200 (Nsp3~Nsp4), 7800~9000 (Nsp9), and 12,200~14,800 (ORF2~ORF6). This study enriches the epidemiological data of PRRSV in northern China, thereby providing theoretical references for the prevention and control of PRRSV in northern China.

## 1. Introduction

The porcine reproductive and respiratory syndrome (PRRS) virus (PRRSV) has caused significant economic losses to the pig industry, as it has rapidly spread worldwide since its discovery in 1987 [1]. PRRSV is characterized by reproductive disorders in sows and piglet respiratory diseases. It is a capsular single-stranded positive-stranded RNA virus with a genome length of approximately 15 kb, encoding eight structural proteins and at least fourteen non-structural proteins (Nsp) [2]. Based on differences in the viral genome sequence, PRRSV can be categorized as *Betaarterivirus europensis* (PRRSV-1) and *Betaarterivirus americense* (PRRSV-2) [3]. The *ORF5* gene is one of the most frequently mutated ORFs in the PRRSV genome, and PRRSV-2 was classified into nine lineages and thirty-seven sub-lineages based on the evolutionary relationship of PRRSV *ORF5* in 2010, with genetic distances of approximately 10–17% between each lineage [4].

Currently, the Chinese epidemic strains of PRRSV-2 include Lineages 1, 3, 5, and 8 [5]. In 1995, the first outbreak of PRRSV in China was reported. In 1996, Strain CH-1a, a representative strain of Lineage 8.1 (L8.1, also known as the classical PRRSV (C-PRRSV)), was isolated for the first time [6]. BJ-4, which showed a high homology with VR-2332, was isolated in 1997 [7]. However, strains of Lineage 5 (L5) represented by BJ-4 were occasionally detected and were not responsible for a widespread epidemic. A highly pathogenic PRRSV (HP-PRRSV) outbreak occurred in China in 2006, characterized by high fever, morbidity, and mortality. For example, JXA1 and HUN4, representative strains of Lineage 8.7 (L8.7), showed 30 discontinuous amino acid deficiencies in the coding region of non-structural protein 2 (Nsp2) [8]. In 1991, a QYYZ-like (representative strain of Lineage 3) PRRSV strain was reported in Taiwan; in 2004, similar strains were reported in Hong Kong; in 2010, such strains were prevalent in southern China [9]. In 2013, a NADC30-like strain, a representative strain of Lineage 1.8 (L1.8), was introduced into China. This strain showed 131 discontinuous amino acid deficiencies in the Nsp2 coding region, revealing high homology to the 2008 US isolate NADC30. This strain mutated rapidly and frequently recombined with other PRRSV wild-type or vaccine strains [10], replacing progressively HP-PRRSV as the predominant prevalent strain in China [11]. In 2014, NADC34, a representative strain of Lineage 1.5 (L1.5), was reported in the US, resulting in severe sow miscarriage and piglet death [12]. Two NADC34-like genotypes were identified in Liaoning in 2018, and they are becoming prevalent in various regions of China [13]. In Nsp2, NADC34 showed 100 continuous amino acid deficiencies, as indicated by the gene identification.

PRRSV is one of the most evolving RNA viruses, with viral antagonism to the host’s innate defenses and the replicase fidelity of the viruses contributing to the enhanced diversity and complexity of PRRSV strains in the field via gene mutation and recombination [14]. PRRSV has experienced intra-lineage recombination, wild-type, and vaccine strains recombination, and mixing and recombination of multiple strains [15,16,17]. Furthermore, the prevention and control of PRRS were significantly impacted by the emergence of a new sub-lineage every 1–4 years [18]. Therefore, it is crucial to establish targeted control strategies and to consistently monitor the dynamic changes in epidemic strain in pig herds. In this study, *ORF5* gene analysis and whole-genome sequencing were performed on suspected PRRSV-positive samples collected in northern China (Hebei, Shanxi, and Liaoning) from 2021 to 2023 to examine the genetic evolution and characteristics of PRRSV, thereby providing a scientific foundation for effective prevention and control strategies in northern China.

## 2. Materials and Methods

### 2.1. Sample Collection

Diagnosis and monitoring of PRRSV were conducted in Hebei, Shanxi, and Liaoning Provinces in northern China between 2021 and 2023. A total of 12,299 clinically suspected PRRSV-positive samples were collected from pig farms of different sizes. Among them, 57.07% of the samples were regularly collected each month from pig farms with over 2000 sows, with 21.46% collected randomly from pig farms with 500–2000 sows and 20.65% collected randomly from farms only involved in fattening pigs, while the remainder were collected randomly from pig farms with fewer than 30 sows. Different sample types were collected according to the type of pig population and the clinical symptoms (see Table 1), including sera, oral fluids, oropharyngeal swabs, tissue samples (including aborted fetuses), piglet processing fluids, and boar semen. These samples were labeled based on their source and collection period and preserved at −80 °C. Approximately 4547 samples were obtained from Hebei, 3799 from Shanxi, and 3953 from Liaoning.

### 2.2. Sample Processing and Analysis

Total RNA was extracted from the samples using a MagaBio-Plus Virus DNA/RNA purification kit (Bori Technology Co., Ltd., Hangzhou, China), according to the kit instructions, and was analyzed using a commercial PRRSV fluorescent quantitative reverse-transcription PCR (qRT-PCR) detection kit (Nabai Biotechnology Co., Ltd., Beijing, China). Data are expressed as the mean ± standard deviation (SD). Statistical analyses were conducted using GraphPad Prism 6.0 software. One-way analysis of variance (ANOVA) was employed to evaluate the differences in the positive rate of PPRSV across various regions and seasons. A *p* value < 0.05 was considered statistically significant.

### 2.3. ORF5 Sequencing

RNA was extracted from 2 to 5 samples that showed positive qRT-PCR detection and Ct values lower than 28 from each pig farm and was then reverse-transcribed to cDNA using a kit (Novozan Biotechnology Co., Ltd., Nanjing, China). The cDNA was used as a template for amplification using *ORF5* gene amplification primers [19] (Supplementary Table S1). To avoid repeated amplification and sequencing of the same strain, samples were sequenced after 6 months in the same pig farm. PCR product purification kit instructions were followed to purify and recover the amplified products, which were then cloned into the pGM-T vector; after being transferred into TOP10 competent cells (Tiangen Biochemical Technology Co., Ltd., Beijing, China), at least three positive clones were selected and sent to Genewiz Biotechnology Co., Ltd., Tianjin, China for sequencing. The *ORF5* gene was assembled in DNAStar 7.0 using SeqMan.

### 2.4. Analysis of ORF5 Gene Phylogeny and Amino Acid Mutation

The sequence of the *ORF5* gene was subjected to multiple sequence alignment using DNAStar 7.0 with CLUSTALW in MegAlign(version 7.1.0). Sequences that showed 100% to other sequences from the same farms were excluded from the analysis, resulting in a final total of 114 *ORF5* gene sequences (Supplementary Table S2). A total of 60 PRRSV *ORF5* sequences (Supplementary Table S3) obtained between 2021 and 2023 from Hebei, Shanxi, and Liaoning Provinces with clear markers, as well as 14 representative strains of different lineages (Supplementary Table S4) were retrieved from the NCBI database. After multiple sequence alignment using MegAlign, the phylogenetic tree of the *ORF5* gene was constructed using the neighbor-joining (NJ) method in MEGA7, with 1000 bootstrap replicates to evaluate branch support. The tree was visualized using Evolview 3.0, with strains of different lineages distinguished by different colors and gene sequences from different sources indicated by different shapes. Finally, variants in the *ORF5* genes from different strains were examined using MegAlign and the online tool WebLogo 3: Create was used to visualize the variants in the protein sequences.

### 2.5. Virus Isolation and Whole-Genome Sequencing

Positive lung tissues with Ct values below 28 from different pig farms in different regions were selected. The tissues were homogenized by grinding and the supernatants were filtered through 0.22 μm filters and inoculated into PAMs that exhibited optimal growth conditions, with a confluence rate of 80% to 100%. After incubation for 2 h at 37 °C with 5% CO_2_, the culture media were aspirated and RPMI 1640 medium containing 2% fetal bovine serum was added, followed by continued culture for 3–5 d. The virus underwent three passages in the cells, and the culture supernatants were collected for extraction of total RNA. The PRRSV qRT-PCR-positive cell culture supernatants were added to PAMs for indirect immunofluorescence assays (IFAs). The primary antibody was a mouse monoclonal antibody against the PRRSV N protein (provided by the Animal Medical College of China Agricultural University), used at a dilution of 1:500, while the secondary antibody was FITC-labeled goat anti-mouse IgG (Abcam, Cambridge, UK), used at a dilution of 1:1000. The identified virus was collected and total RNA was extracted and reverse-transcribed to cDNA, which was used as a template. The whole-genome sequence of PRRSV was amplified using 12 pairs of specific primers [19] (Supplementary Table S1), followed by sequencing and assembly as described in 1.3.

### 2.6. Recombination Analysis

The whole-genome sequences of 17 PRRSV strains from different farms in different regions were obtained through isolation and sequencing. The complete genomes of 10 PRRSV-2 strains (length > 15 kb) obtained from the Hebei, Shanxi, and Liaoning Provinces from 2021 to 2023 were downloaded from NCBI. Six representative strains, namely, NADC34 (L1.5), NADC30 (L1.8), QYYZ (L3.5), VR2332 (L5.1), CH-1a (L8.1), and JXA1 (L8.7), were selected as reference parents. Recombination events were preliminarily detected using SimPlot V3.5 software, with a window size of 200 bp and a step size of 20 bp, and the recombination parent strain and breakpoint location were determined. Then, seven recombination algorithms in Recombination Detection Program v.4.24 (RDP 4.24), including the RDP, GENECONV, Maxchi, Chimaera, 3Seq, Bootscan, and SiSscan programs, were used to detect and analyze possible recombination events. Only when the recombination event was detected by at least four of the seven programs and the *p*-value was ≤0.01 was it considered that recombination may have occurred.

## 3. Results

### 3.1. Clinical Sample Detection and Analysis

**Figure 1:**
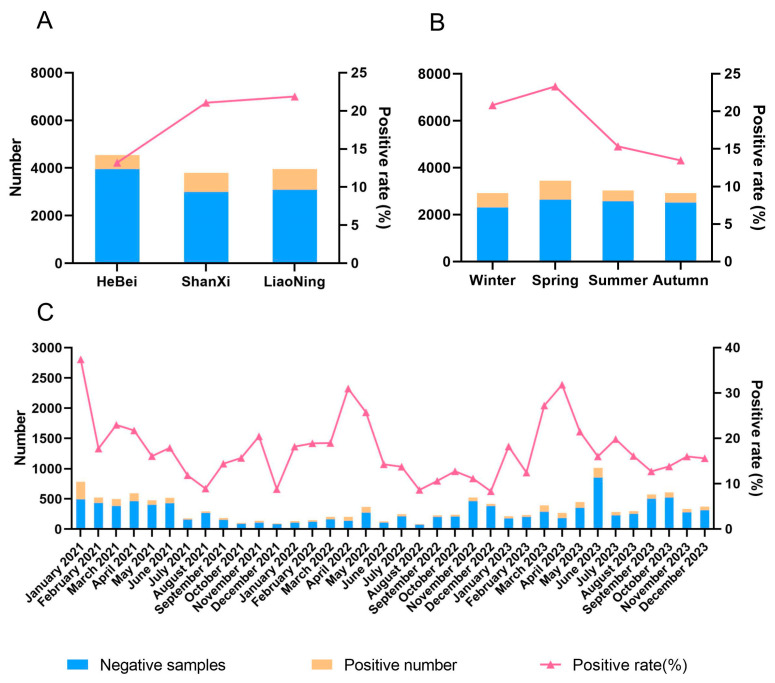
Positivity rate of PRRSV in northern China during 2021–2023. Note: (**A**): positivity rates of PRRSV in different regions; (**B**): positivity rates of PRRSV in different seasons; (**C**): positivity rates of PRRSV in each month during 2021–2023.

A total of 12,299 clinically suspected PRRSV-positive samples collected in Hebei, Shanxi, and Liaoning during 2021–2023 were investigated by qRT-PCR. As indicated, 2266 samples were PRRSV-positive, with a positive rate of 18.42% (2266/12,299). As shown in Figure 1A and Figure S1, the positive rate of PRRSV in Hebei (13.20%) was lower than that in Shanxi (21.08%) and Liaoning (21.88%). As shown in Figure 1C, the positive rate of PRRSV was relatively high in January 2021, April 2022, and April 2023; the incidence rates of PRRS in spring (March–May), summer (June–August), autumn (September–November), and winter (December–February) were 23.31%, 15.33%, 13.47%, and 20.82%, respectively (Figure 1B and Figure S2), with spring being significantly higher than summer and autumn (*p* ≤ 0.05).

### 3.2. Analysis of ORF5 Gene Phylogeny

**Figure 2:**
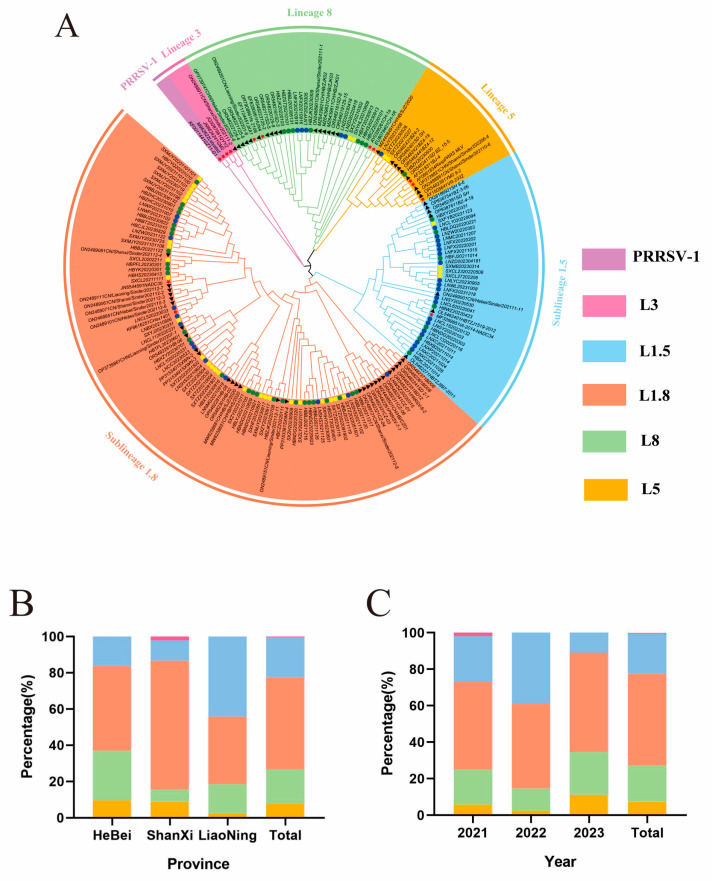
Classification of PRRSV lineages in northern China during 2021–2023. Note: (**A**): phylogenetic tree based on *ORF5* gene sequences; red asterisks are representative strains of different lineages, black triangles are NCBI downloaded strains, and different colored circles are strains from different provinces; (**B**): percentage of different lineages in each province; (**C**): proportion of different lineages in different years.

The *ORF5* genes of 114 sequenced strains in this study (Supplementary Table S2) and 60 PRRSV strains downloaded from NCBI (Supplementary Table S3) were used to construct a phylogenetic tree using MEGA7. Figure 2A illustrates that all 174 *ORF5* gene sequences were classified as PRRSV-2, with 88 corresponding to L1.8 (50.57%), 38 to L1.5 (21.84%), 34 to L8 (19.54%), 13 to L5 (7.47%), and 1 to L3 (0.57%). The strain types were diverse across regions. In Hebei and Shanxi, the proportion of L1.8 was higher than that of other lineages, at 46.51% and 71.11%, respectively (Figure 2B). In Liaoning, the proportion of L1.5 was higher than that of other lineages, at 44.19%. During the three years, the proportion of various strain types varied slightly, with an increase in the proportion of L1.8, L8, and L5, and a decrease in the proportion of L1.5 (Figure 2C).

### 3.3. Mutational Analysis of ORF5 Amino Acid Sequences

**Figure 3:**
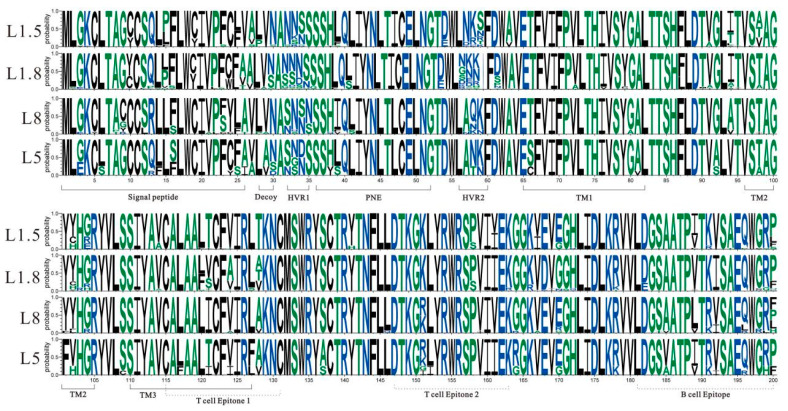
Diversity analysis of amino acids of PRRSV-2 *ORF5* in northern China during 2021–2023. Note: The height of each amino acid letter in the sequence logos corresponds to its frequency in all the sequences from the corresponding subgroup.

The amino acid mutation sites of 114 sequenced *ORF5* genes were examined, as shown in Figure 3, revealing no insertions or deletions of amino acids in L5, L8, and L1.5. In L1.8, there were 6 out of 66 insertions of N^60^ amino acids at position aa60, 1/66 strains showed deletions of N^33^ and D^34^ amino acids, 1/66 strains had deletions of D^34^ amino acids, and 1/66 strains had deletions of S^36^ and S^37^ amino acids. Except for L8, other lineages with a total of 100 amino acids of *ORF5* genes did not match the characteristics of strong strains of *ORF5* proteins R^13^ and R^151^; except for L5, other lineages did not match the characteristics of vaccine strain A^137^ [20].

As indicated in Figure 3, the decoy epitopes (A^27^/V^27^L^28^V^29^N^30^/S^30^) of the *ORF5* protein associated with immune responses were polymorphic at sites A^27^/V^27^, L^28^, and N^30^/S^30^. For L1.5, 2/30 mutations were A^27^/V^27^ → S^27^, while no mutations were found in other lineages. For L1.5 and L8, 6/30 and 1/14 mutations were L^28^ → P^28^, respectively; for L1.8, 2/66 mutations were L^28^ → F^28^; for L5, 1/4 mutations were N^30^/S^30^ → D^30^; for L1.5, 1/30 mutations were N^30^/S^30^ → D^30^; for L1.8, 2/66 mutations were N^30^/S^30^ → G^30^. In the neutralized antigenic epitopes (S^37^H^38^F^39^/L^39^Q^40^L^41^I^42^Y^43^N^44^) of the *ORF5* protein associated with immune responses, several strains were mutated at L^39^ and L^41^. L8 had 13/14 mutations of L^39^ → I^39^ and 1/14 mutations of L^39^ → V^39^; and L1.8 had only 1/30 mutations of L^39^ → S^39^. L8 and L1.5 had 2/14 and 16/30 mutations of L^41^ → S^41^, respectively.

Both the T- and B-cell epitopes of the *ORF5* protein were examined (Figure 3), revealing changes in specific amino acids across several lineages. L1.5 had two distinct amino acid alterations at T^121^ and T^128^ inside T-cell epitope regions. L1.8 had four unique amino acid mutations in T- and B-cell epitopes, respectively, including S/V^121^, A^124^, D^168^, and G^170^.

### 3.4. Isolation of PRRSV Strains

**Figure 4:**
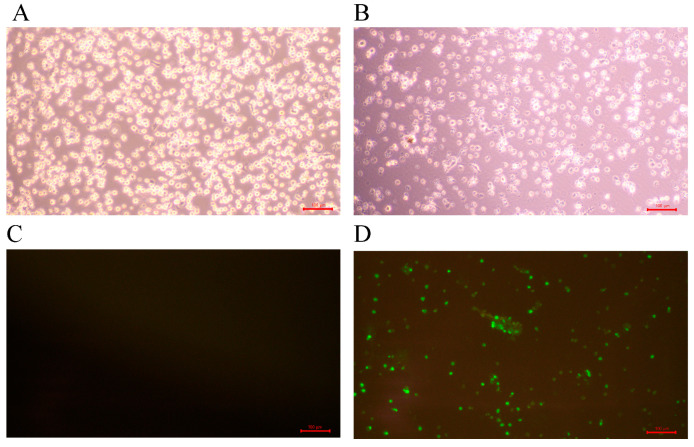
CPE and IFA in PAMs infected with PRRSV isolates (100×). (**A**): negative control cells (white light); (**B**): pathological cells (white light); (**C**): negative control cells (fluorescence); (**D**): pathological cells (fluorescence).

The samples inoculated with PAMs showed visible evidence of cytopathic effects (CPEs), such as cell shrinkage and rupture (Figure 4B). The virus supernatants detected by qRT-PCR were positive for IFA identification and specific green fluorescence was observed on PAMs (Figure 4D), demonstrating the successful isolation of PRRSV with a total of 17 strains.

### 3.5. Whole-Genome Recombination Analysis

**Figure 5:**
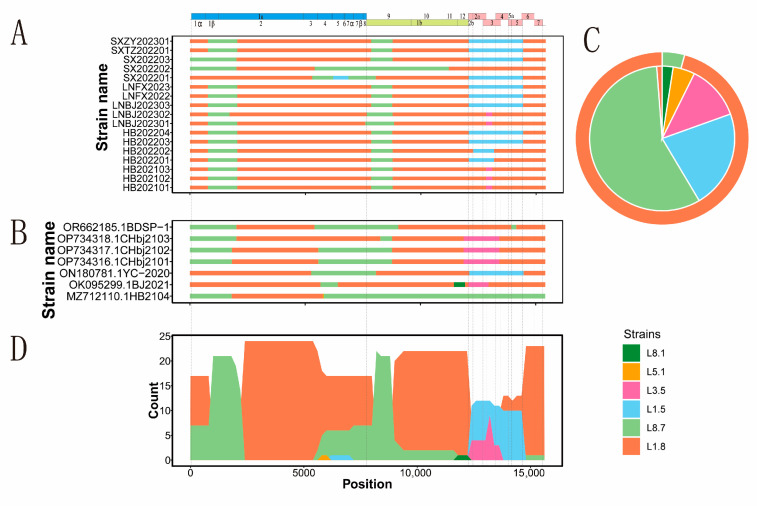
PRRSV-2 genome recombination analysis in northern China during 2021–2023. Note: (**A**): the 17 PRRSV genome recombinant lineages obtained in this study; (**B**): a total of 7 PRRSV genome recombinant lineages downloaded from NCBI; (**C**): proportion of main and secondary potential parental strains of 24 recombinant strains. The proportion of each main potential parental lineage is shown in the outer ring, and the proportion of each potential secondary parental lineage is shown in the pie chart; (**D**): frequency overlay of each lineage strain participating in recombination as potential secondary parents.

A total of 17 strains obtained by sequencing and 10 PRRSV whole-genome sequences downloaded from NCBI were analyzed using Simplot and RDP, respectively. Similarly, 17 strains were recombinant (Figure 5A, Supplementary Table S5), and 7 out of 10 NCBI strains were recombinant (Figure 5B, Supplementary Table S6). L1.8 was the primary parental strain, providing 23 out of 24 strains, which accounted for 95.83% (Figure 5C), indicating that L1.8 dominated by providing the backbone for most recombination events. The main secondary parent was L8.7, followed by L1.5 and L3.5, and a small amount of L5.1. The recombination breakpoint positions were distributed in 780~2200 (Nsp1~Nsp2), 5400~6200 (Nsp3~Nsp4), 7800~9000 (Nsp9), and 12,200~14,800 (ORF2~ORF6) (Figure 5D). There were three primary recombination modes among the 24 strains, as follows: the intra-/inter-lineage recombination mode of L1.8, L8.7, and L1.5 accounted for 50.00%, the inter-lineage recombination mode of L1.8, L8.7, and L3.5 accounted for 37.50%, and the recombination mode for L1.8 and L8.7 accounted for 8.30%. Interestingly, L1.5 and L3.5, as secondary parents, mainly provided GP2, GP3, GP4, GP5, or GP6 structural proteins.

## 4. Discussion

Porcine reproductive and respiratory syndrome virus (PRRSV) is susceptible to mutation and recombination. It has been the source of three epidemics since its introduction to China, characterized by the CH-1a, JXA1, and NADC30 strains, significantly complicating the prevention and control of PRRS. In this study, a total of 12,299 clinically suspected PRRS samples were collected in northern China during 2021–2023, and the PRRSV detection rate was 18.42% (2266/12,299), which was lower than the positivity rate (26.07%) reported by Wu [21] in a survey of 27 provinces in China during 2017–2018, and lower than the positivity rate of 34.8% (175/503) reported by Li [22] in four provinces of northern China from 2016 to 2018. This may be attributable to the 57.07% of samples obtained from large-scale pig farms with over 2000 sows that consistently enhanced their biosecurity measures in response to the effects of African swine fever. For example, personnel, materials, and vehicles could only enter the pig farm after multiple rounds of cleaning and disinfection. Pigs newly introduced to the farm were only allowed if they tested negative for both African swine fever and PRRSV nucleic acids. The large-scale pig farms successively increased their air-filtration equipment. These measures played a role in reducing the spread and rate of PRRSV infection.

Low temperatures and significant temperature fluctuations may intensify the dissemination of PRRSV, rendering pigs more vulnerable to respiratory diseases. In the regional comparison, the detection rate of PRRSV was higher in Liaoning (21.88%) and Shanxi (21.08%) than in Hebei (13.20%), where temperatures were relatively high. Liaoning Province is located in the northeastern region of China. The annual average temperature is 3 °C–15 °C, and the average temperature in winter is −5.7 °C. Shanxi Province is located in northern China. The annual average temperature is between 3 °C and 14 °C, and the average temperature in winter is −2.7 °C. In North China, Hebei Province has an average annual temperature range of 10 °C–20 °C. Low temperatures and the long-term low-temperature maintenance of PRRSV may increase the spread and infection rate of PRRSV by prolonging its survival time and reducing the efficacy of disinfectants. Observing the seasonal changes, spring (23.31%) and winter (20.82%) had higher detection rates than summer (15.33%) and autumn (13.47%). Notably, the rate in spring was significantly higher than in summer and autumn (*p* ≤ 0.05). This may be related to the specific conditions seen during spring and winter, when large temperature differences between day and night increase the likelihood of respiratory diseases. In addition, factors such as breeding density, scale, and mode in the region can also affect the spread of PRRSV. Since the change introduced in the breeding mode in northern China after African swine fever, more data need to be collected for further analysis.

Wu [23] examined 1439 PRRSV-2 *ORF5* sequences in GenBank (including 34 PRRSV *ORF5* determined by the author) collected between 2010 and 2021 from all over China, revealing a progressive increase in the proportion of L1, which exceeded L8 in 2016 and reached 92.75% (64/69) by 2021, identifying it as the predominant epidemiological strain. In this study, 114 *ORF5* gene sequences were obtained, 60 *ORF5* gene sequences were downloaded from three provinces for genetic evolutionary analysis, and 174 PRRSVs were found to be PRRSV-2. The proportions of L1.8, L1.5, L8, L5, and L3 were 50.00%, 21.84%, 20.11%, 7.47%, and 0.57%, respectively. This suggested that the main PRRSV epidemic strain in northern China was L1.8, followed by L1.5 and L8. In 2017, the first L1.5 strain in China was found in Liaoning and then rapidly developed in North China, East China, Central China, South China, and Southwest China. Xu et al. [24] collected 433 positive clinical samples from all over the country between 2020 and October 2021, and comprehensively evaluated the phylogenetic, epidemic, and recombination characteristics of NADC34-like PRRSV in China. The results indicated that the prevalence of L1.5 increased from 3% between 2017 and 2019 to 28.60% in 2021, identifying it as one of the predominant strains extensively distributed in specific regions of China. In this study, it was identified that the proportion of L1.5 was higher in Liaoning (44.19%), whereas in Hebei and Shanxi, the proportions of L1.5 (16.28% and 11.11%) were lower than that of L1.8 (46.51% and 71.11%). During 2021 and 2023, L1.8 and L8 had an upward trend, whereas L1.5 demonstrated a declining trend. Therefore, it is necessary to continue strengthening the monitoring of PRRSV strains.

PRRSV GP5 protein, as the key structural protein for viral invasion and induction of host production of neutralizing antibodies, is an important target for analyzing the genetic mutation and antigenic epitopes of PRRSV. In this study, the amino acids of 114 *ORF5* genes were mutated in decoy epitopes, neutralizing epitopes, and B- and T-cell epitopes. Especially, L1.8, L1.5, and L8 were significantly polymorphic at amino acid sites A^27^/V^27^L^28^V^29^N^30^/S^30^ and S^37^H^38^F^39^/L^39^Q^40^L^41^I^42^Y^43^N^44^. These mutations and variations enhanced the viral immune escape ability by affecting the decoy effect and immunogenicity of neutralizing epitopes [25]. This may be one of the significant driving factors for the dominance of L1.8, L1.5, and L8 within the epidemic strains. Strains of different lineages have unique amino acid mutations on T- and B-cell epitopes. For instance, L1.5 has two unique amino acid mutations (T^121^ and T^128^), and L1.8 has four unique amino acid mutations (S/V^121^, A^124^, D^168^, and G^170^). However, the correlation of unique amino acid mutations with virulence and immunogenicity of strains of different lineages remains unclear. PRRSV *ORF5* aa13 and aa151 are closely related to virulence [26]. The amino acids of the 100 *ORF5* genes in this study, except for L8, did not conform to the characteristics of the strong virulent strains of R^13^ and R^151^, suggesting that the current PRRS strains were possibly evolving towards a more moderate direction of virulence, but the observed ease of recombination in the PRRSV gene cast some doubt on this [27].

Gene recombination is regarded as a crucial mechanism for the emergence of new strains, and the incidence of PRRSV recombination has been expedited by large-scale, high-density breeding, vaccination practices, and the widespread presence of various strains on swine farms. In this study, all 17 whole-genome sequences obtained were exposed to recombination, further demonstrating that PRRSV recombination events were frequent and the dominant epidemic PRRSV strains in northern China were recombinant ones. The diversity of recombination modes may lead to differences in PRRSV cellular tropism and pathogenicity, e.g., the pathogenicity of recombinant strain FJ1402 of L1 and L8 was higher than that of L1.8 and recombinant strains of L5 strains [28]. The PRRSV recombination mode in this study was extensively analyzed, and it was found that L1.8 was the primary source of recombinant strains. The recombination mode was primarily a combination of L1.8 + L8.7 + L1.5/L3.5, indicating that L1.8 and L8 played an increasingly significant role in recombination interactions. In recombination events, L1.5 and L3.5 mainly provided GP2, GP3, GP4, GP5, or GP6 structural proteins, which were associated with the PRRSV cellular tendency and replication and had important impacts on PRRSV survival, transmission, and even pathogenesis.

The PRRSV recombination hotspots were investigated, and the results showed that recombination hotspots were concentrated in 780~2200 (Nsp1~Nsp2), 5400~6200 (Nsp3~Nsp4), 7800~9000 (Nsp9), and 12,200~14,800 (ORF2~ORF6). These results were similar to those reported by Yu et al. [11]. Cui et al. [29] analyzed the AU content, secondary protein structures, protein localization, and RdRp codon preference of these recombination hotspots. They found that RdRp codon usage bias had a more significant impact on recombinant viruses. This provides a new perspective on the recombination mechanism of PPRSV and needs to be analyzed in depth in the future.

Overall, the epidemic strains of PRRSV in northern China are distinguished by their complexity and diversity. However, some patterns can be observed, which will aid in understanding the epidemiological trend and recombination mode in northern China, evaluating or developing strategies for the prevention and control of PRRSV, and identifying a safe strategy for developing a new vaccine.

## 5. Conclusions

In this study, the genetic evolution of PRRSV in northern China was analyzed. The results showed a PRRSV positivity rate in northern China of 18.42%. The most prevalent epidemic strain was L1.8, followed by the L1.5 and L8 strains, with most being recombinant strains. The mode of recombination was largely a combination of L1.8 + L8.7 + L1.5/L3. These findings provide more epidemiological data on PRRSV in northern China, which is helpful for the prevention and control of PRRSV in this area.

## Figures and Tables

**Table 1:** Sampling strategies.

Age of Piglet	Type of Sample	Sampling Time	Sample Size
Boars	Semen/oropharyngeal swab	Quarterly	20% of each batch with pools of 5
Sows	Serum/tissue samples	When abortion occurs	Each pig
Suckling piglets	Processing fluid	Each batch of piglets	Pools of 20–30 litters
Weaned piglets	Serum/oropharyngeal swab	3 days before and after weaning	Weak pigs with pools of 5
Fattening pig	Serum/oral fluid	Suspected clinical symptoms	Pools of 15–20 litters
Dead pigs	Tissue samples	Immediately	Each pig

## Data Availability

All the data underlying the findings described in this article are available within the article. For any data not included in the article, please contact the corresponding authors.

## Supplementary Materials

The following supporting information can be downloaded at: https://www.mdpi.com/article/10.3390/v17010085/s1: Table S1: PCR primer information; Table S2: The list of 140 *ORF5* nucleotide sequences with corresponding information; Table S3: The list of 60 *ORF5* nucleotide sequences downloaded from NCBI; Table S4: The list of reresentative strains of 14 different lineages downloaded from NCBI; Table S5: The list of 17 whole-genome sequences of PRRSV with corresponding information; Table S6: The list of 10 whole-genome sequences of PRRSV downloaded from NCBI with corresponding information. Figure S1: Positivity rates of PRRSV in different regions; Figure S2: Positivity rates of PRRSV in different seasons.
