# Supplementary figures and images for: Molecular Epidemiology and Genetic Evolution of Porcine Reproductive and Respiratory Syndrome Virus in Northern China During 2021–2023
Source: Viruses. 2025 Jan 11;17(1):85. doi: 10.3390/v17010085 (PMC11769476; doi:10.3390/v17010085)

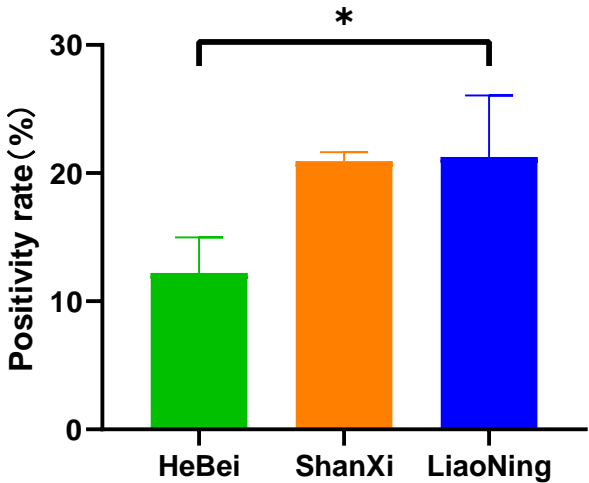

Supplement: Supplementary file 1 [file viruses-17-00085-s001.zip › Figure S1. Positivity rates of PRRSV in different regions.pdf]

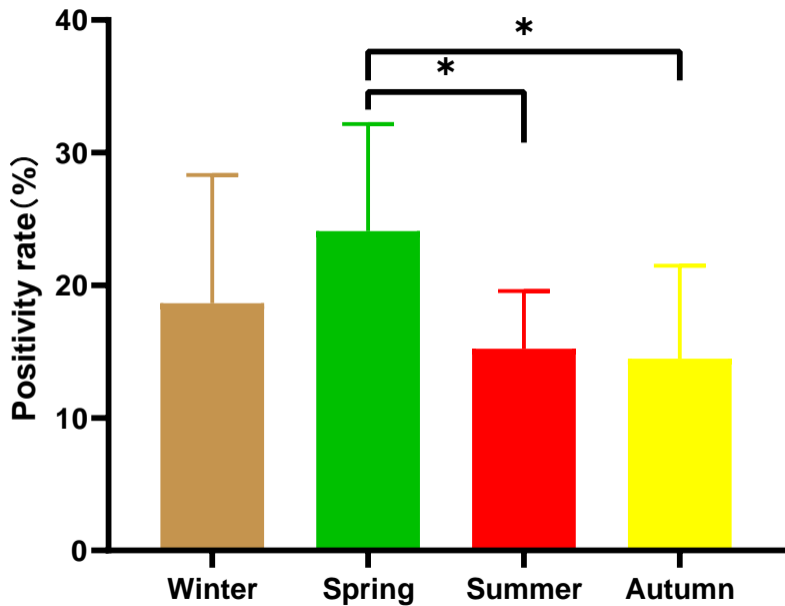

Supplement: Supplementary file 1 [file viruses-17-00085-s001.zip › Figure S2.positivity rates of PRRSV in different seasons.pdf]
